# Probing the effect of ethylene carbonate on optimizing the halogen-free electrolyte performance for Mg sulfur batteries

**DOI:** 10.1039/d3ra03985d

**Published:** 2023-07-13

**Authors:** Mostafa. A. Moselhy, Mohamed Farrag, Yujie Zhu, Eslam Sheha

**Affiliations:** a Physics Department, Faculty of Science, Benha University 13518 Benha Egypt islam.shihah@fsc.bu.edu.eg; b School of Chemistry, Beihang University Beijing P. R. China

## Abstract

Magnesium metal batteries attract great attention for their high volumetric capacity and safety as a post-lithium choice. The strategy of adding organic plasticizer may bring new insights into designing halogen-free electrolytes for the further development of magnesium–sulfur batteries. The high charge density of Mg^2+^ results in a high desolvation barrier and low interfacial Mg^2+^ transfer kinetics due to the strong coulombic interactions of Mg^2+^ ions with anions and solvent molecules. In this study, we test the effect of the stoichiometric ratio of ethylene carbonate (EC) as an organic additive on the electrochemical performance of halogen-free electrolyte (HFE) based on Mg(NO_3_)_2_ in acetonitrile (ACN) and tetraethylene glycol dimethyl ether (G4). Through various characterization methods, the introduction of EC perturbs the bonding scheme of the HFE electrolyte, enhances the ionic conductivity, reduces the relaxation time, and forms a resistive solid electrolyte interphase (SEI). The assembled Mg–S full cell using modified HFE (HFE_EC) delivers initial specific capacities of 900 m Ag^−1^ with a cycle life of up to 10 cycles in the case of activating the cell with electrochemical conditioning. This study sheds light on the interplay of EC and the interfacial kinetics in Mg batteries and opens a door for designing novel magnesium electrolytes.

## Abstract 2

Ethylene carbonate perturb the bonding scheme of HFE and catalyze the dissociation of the dissolved salt and hence electrolyte conductivity.

## Graphical abstract

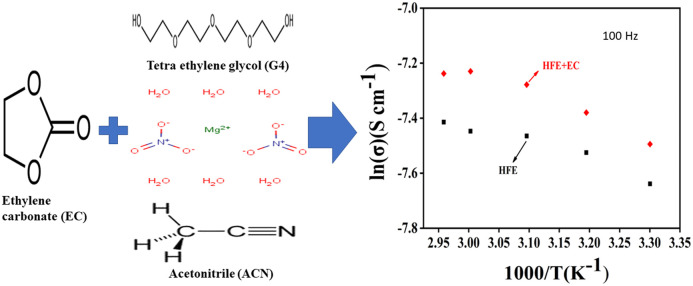

## Introduction

The transition from fossil fuel to green fuel depends on the ability to bridge the gap of renewable energy intermittency through sustainable energy storage systems. Mg–S batteries are an attractive option due to their low cost, environmental sustainability, and abundant magnesium and sulfur in the Earth's crust. In addition, Mg–S batteries offer competitive theoretical energy density (1684 W h kg^−1^ and 3286 W h L^−1^) and safety (dendrite less) over their Li counterparts. However, there are technical challenges like the poor cyclability, the shuttle effect of polysulfides, and the highly resistive passive anode/electrolyte interface that must be overcome before the practical transition from the lab to the market.^1^ BaTiO_3_ possesses a piezoelectric property, generating electrostatic charges on the surface under electrochemical stress. This property gives BaTiO_3_ the ability to immobilize the polar polysulfide molecules *via* chemical adsorption, reducing polysulfide shuttling and improving the cycle life of Mg–S cells.^2,3,4^ The electrolyte controls the electrochemical activity, safety stability, and thermodynamics of the batteries. Therefore, designing an efficient magnesium electrolyte compatible with sulfur's electrophilic nature will be essential to achieving a practical Mg–S battery. In this regard, non-nucleophilic electrolytes were designed, including hexamethyldisilazide-Br,^5^ non-nucleophilic Mg(TFSI)_2_–MgCl_2_–1,2-dimethoxyethane/tetrahydrofuran,^6^ magnesium tetrakis(hexafluoroisopropyloxy)borate,^7^ magnesium perfluorinated *tert*-butoxide (Mg(pftb)_2_),^8^ Mg[B(Ohfip)_4_]_2_.^9^ Nevertheless, the common factors of the most weakly coordinating Mg salts are the low stability, high cost, and complex synthesis process, that impede their practical applications. In our previous research,^10^ we reported a simple halogen-free electrolyte (HFE) 0.69 M Mg(NO_3_)_2_·6H_2_O in ACN : G4 (∼2 : 1) with ionic conductivity of ∼10^−4^ S cm^−1^ at 313 K, dendrite-free, and translucent Mg deposits. However, much work need to be dedicated to lowering the Mg^2+^ desolvation energy and reducing the Mg stripping/plating overpotential. Ethylene carbonate (EC) organic cosolvent is commonly used to tailor the interfacial kinetics and the transport properties of the electrolyte and played a vital role in the commercialization of lithium-ion batteries. The high dielectric constant of EC grants it the ability to dissociate metal salt and engineer the solid anode/electrolyte interphase.^11,12^ Herein, we study for the first time the beneficial effects of the interplay between HFE and EC additive and how it affects the Mg^2+^ desolvation energy. The study was revealed by UV-vis spectroscopy, Fourier transforms infrared (ATR-FTIR) spectroscopy, and electrochemical impedance spectroscopy (EIS). Galvanostatic cycling was conducted on the Mg/Mg symmetric battery with the HFE_EC electrolyte to test the change of the overpotential with introduction of EC. The nature of Mg/HFE_EC interface was probed using FTIR, X-ray diffraction (XRD), and SEM_EDS techniques. Mg–S cells with HFE and HFE_EC electrolytes were assembled and tested electrochemically, and the sulfur cathode was retrieved from the disassembled cells to examine the morphology and structure of the S cathodes at different electrochemical states using SEM, EDX, XRD and EIS. The study confirms the impact of EC additive to regulate the electrochemical performance of the HFE *via* modulating Mg^2+^ desolvation barrier but it results in undesirable SEI which causes high Mg stripping/plating overpotential.

## Experimental work

The halogen-free electrolyte (HFE) based on 0.69 M Mg(NO_3_)_2_·_6_H_2_O dissolved in ACN : G4 (∼2 : 1) was synthesized according to the published work.^10^ Five bottles of HFE were prepared and *x* M of EC, (*x* = 0, 0.05, 0.1, 0.15, 0.2) was added to each one, which were denoted as HFE, HFE_X_1_, HFE_X_2_, HFE_X_3_, and HFE_X_4_ respectively. ATR-FTIR measurements were recorded in the wavenumber ranging from 400 to 4000 cm ^−1^ using ALPHA II Bruker spectrometer. UV-vis spectral data were collected using Edinburgh DS5 Dual Beam UV-vis spectrophotometer. The S cathode was prepared by grinding 75 wt% of sulfur (S, Alfa Aesar 99%) with 20 wt% graphene nanoplatelets (GNPs, Grade M, XG Science), and 5 wt% BTO (Alfa Aesar 99%). The resultant mixture was placed in the microwave for 10 seconds and then subjected to a ball mill for 30 hours to obtain the S_GNP_BTO active cathodic material. 0.75 g S_GNP_BTO : 0.1 g Super C^P^ : 0.15 g polyvinylidene fluoride was dissolved in *N*-methyl-2-pyrrolidinone using a magnetic stirrer. The resultant viscous slurry was spread on Al foil with a thickness of 100 μm using HOHSEN MC-20 Mini-Coater and left in the oven for two hours at 100 °C. After that, the cathode was cut with a diameter of 14 mm and then permanently kept at a temperature of 65 °C. Electrochemical impedance spectroscopy (EIS) and electrochemical measurements of the electrolyte were tested using CHI604E electrochemical workstation. The collected impedance data were analyzed by ZView software. The galvanostatic test was conducted between 0.25 and 2.5 V using a NEWARE BTS4000. XRD pattern was recorded on Rigaku MiniFlex 600 diffractometer, while SEM images were captured using Jeol JMS-700 EDS electron microscope.

## Results and discussions

**Fig. 1:**
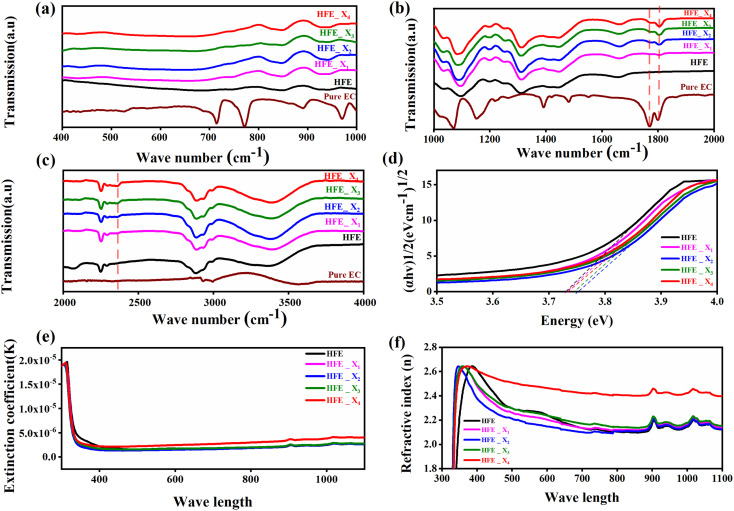
(a, b and c) FTIR spectra, (d) (*αhν*)^0.5^*vs. hν*, (e) *n vs. λ*, (f) *k vs. λ*, HFE, 5%, 10%, 15% and 20%; of HFE__*x*_EC electrolytes.

**Fig. 2:**
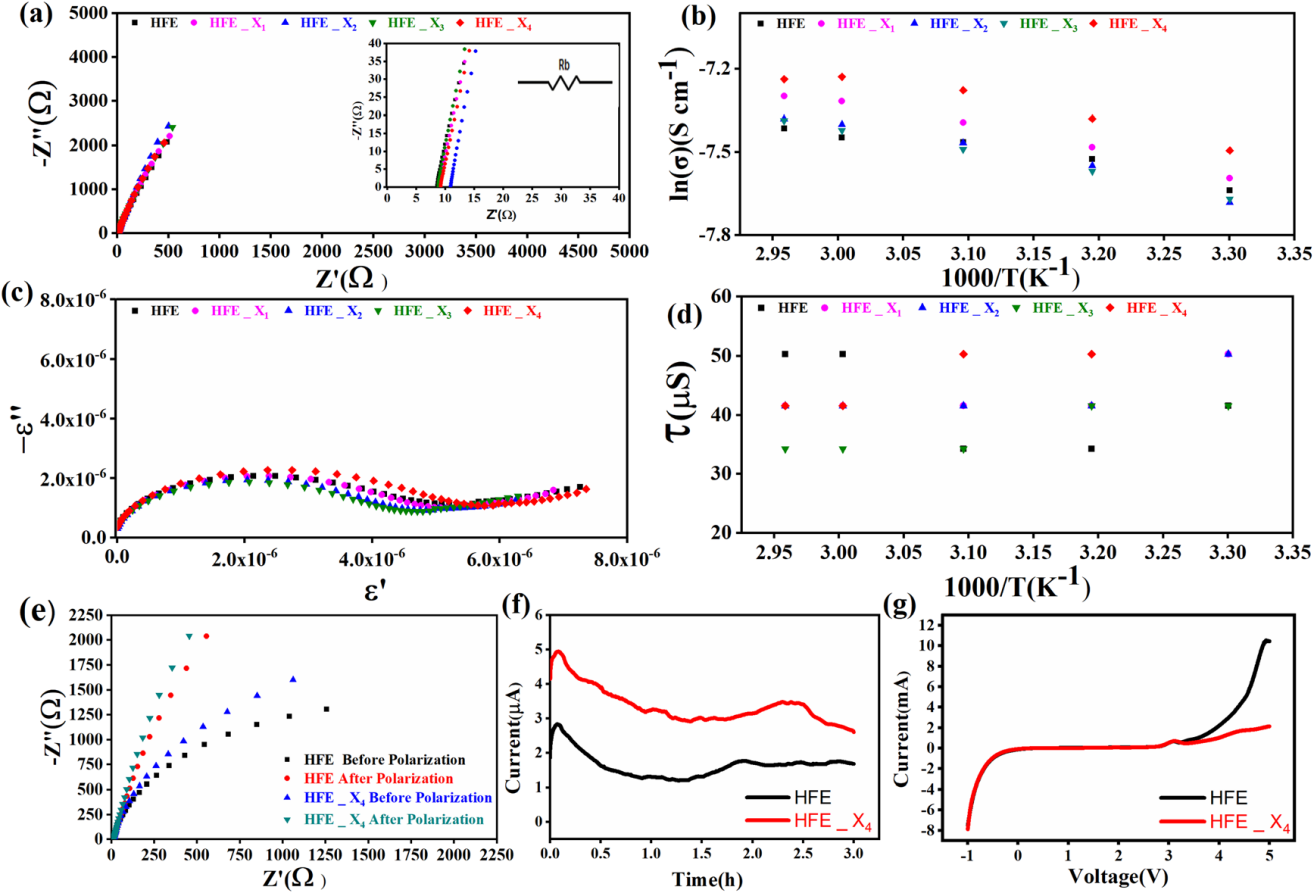
(a) Nyquist plots of a symmetric SS//electrolytes//SS cell at 303 K; inset is the zoom-in intersection of EIS spectra with the *Z*′ axis; (b) Arrhenius plot of *σ* ∼ 10^3^/*T* at 100 Hz; (c) Cole–Cole plots at 303 K; (d) relaxation time *vs.* temperature; (e) Nyquist plots of symmetric Mg//electrolyte//Mg cells before and after polarization at room temperature (f) polarization current curve *vs.* time of symmetric Mg//electrolyte//Mg cells; (g) LSV of Mg//electrolyte//SS cells at room temperature.

The functional group's evolution and possible molecular interaction of HFE__*x*_EC electrolytes can be identified using the FTIR spectroscopic analysis. Fig. 1a–c shows the ATR-FTIR spectra of HFE__*x*_EC electrolytes in the 400–4000 cm^−1^ range. The spectra of the pristine sample exhibit spectral lines that match well with the bands of HFE that have been reported in our previous study.^13^ After EC addition to HFE, there are two bands at 1770 and 1796 cm^−1^ grew up with increasing the content of EC that match well with the C=O stretching mode of pure EC.^14^ Furthermore, the introduction of the EC in the structural skeleton of the HFE results in additional small new peak characteristic of –C≡N⋯Mg^2+^ at wavenumber 2362 cm^−1^, which indicates the role of EC in engineering electrolyte solvation structure *via* supporting weak solvent–cation interaction, promoting the rapid desolvation of Mg^2+^ ions.^15^ The UV-visible absorption spectra are a powerful tool to analyze the band structure of the energy materials. Fig. 1d–f shows the UV-visible absorption spectrum of HFE__*x*_EC electrolytes in the 200–1100 nm range. The band gap energy *E*_g_ was estimated using Tauc's relation:^16^*αhν* = *β*[*hν* − *E*_g_]^*n*^, Fig. 1d displays the direct electronic transition profile of *hν versus* (*αhν*)^0.5^ for HFE__*x*_EC electrolytes. The *E*_g_ estimated from the extrapolation of the linear region of the plot on the *x*-axis (*αhν* = 0) gives the value of the optical bandgap, *E*_g_. The bandgap was found to be around 3.43 eV of the HFE__*x*_EC electrolytes; hence HFE__*x*_EC shows the difficulty in losing electrons (non-nucleophilic), which makes it a compatible electrolyte with the electrophilic nature of the S_8_. Fig. 1e displays the extinction coefficient 
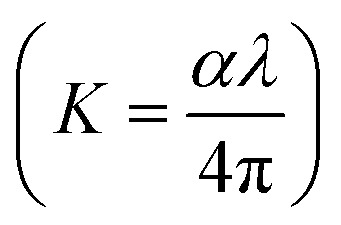
*versus* the wavelength (*λ*) of HFE__*x*_EC electrolytes. The value of *k* shows a high dispersion with increasing wavelength in the UV period (200–340 nm) and is followed by a little increase in the visible region. Also, it can be noticed that the value of *k* relatively increases with increasing EC content. The refractive index (*n*) is an optical property that measures the change in the velocity of light inside the electrolyte, which can be calculated using the equation:^17,18^
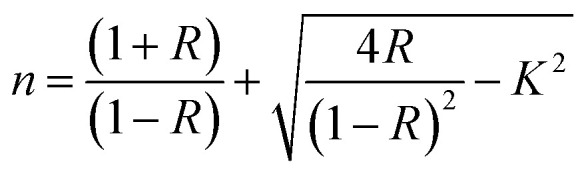
, where *R* = [1−(*A* + *T*)], *A* and *T* are the absorption and transmission coefficient, respectively. Fig. 1f shows the spectra of refractive index *versus* the wavelength (*λ*) of the relation between the refractive index (*n*) of HFE__*x*_EC electrolytes. The profile shows increasing the ability of the HFE__*x*_EC mediums to bend the path of the electromagnetic waves with the transition from UV to the visible regions and increasing EC content. This indicates EC's role in changing the optical density and electronic structures of HFE. Fig. 2a shows the Nyquist plots of symmetric stainless steal (SS)//electrolyte//SS coin cell at 303 K. The spectra show an incomplete semicircle at the high-frequency region representing the bulk resistance *R*_b_ (due to migration of ions) and bulk capacitance (due to immobile species), whereas the low-frequency spike is due to the interfacial polarization effect.^19^ The bulk conductivity was calculated by fitting the semicircle curves using ZView software and substituting in the following eqn: 
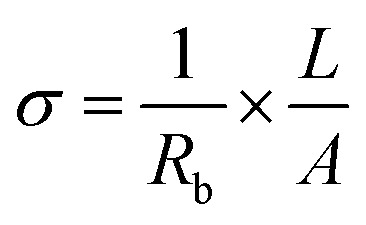
, where *L* and *A* are the thickness and cross-sectional area of separator, respectively. Arrhenius equation: 
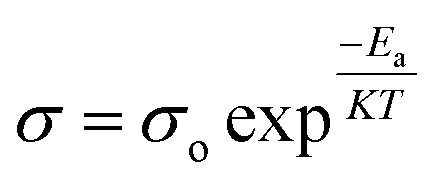
 was used to calculate the activation energies *E*_a_ of ion conduction, Fig. 2b shows the Arrhenius plot 
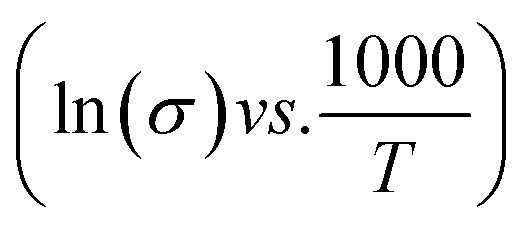
 at 100 Hz of HFE__*x*_EC electrolytes, where *σ*_o_, and *K* represent the pre-exponential conductivity factor, and the Boltzmann constant. The calculated activation energies are 0.052, 0.076, 0.075, 0.07 and 0.067 eV for HFE, HFE_X_1_, HFE_X_2_, HFE_X_3_, and HFE_X_4_ respectively. The behavior of the activation energy with the increasing the content of EC can be understood in the light of the low concentration of EC was not enough to dissociate the ions.

**Fig. 3:**
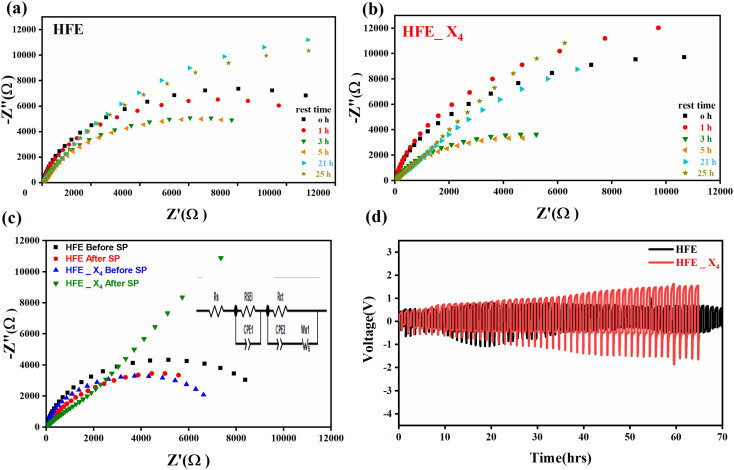
Nyquist plots of a symmetric Mg//electrolytes//Mg cell (303 K) at different storage times for (a)HFE; (b) HFE_X_4_; (c) Nyquist plots of a symmetric Mg//electrolytes//Mg cell before and after SP; (d) stripping//plating of Mg//electrolytes//Mg cells.

**Fig. 4:**
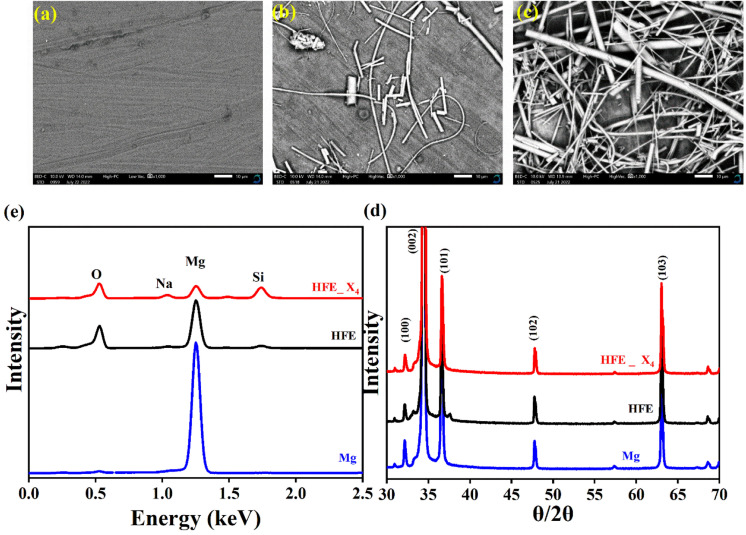
SEM micrographs (a) bare Mg; (b) Mg_HFE; (c) Mg_HFE_X_4_; (d) XRD patterns. (e) EDS spectra of bare Mg, Mg_HFE, and Mg_HFE_X_4_.

**Fig. 5:**
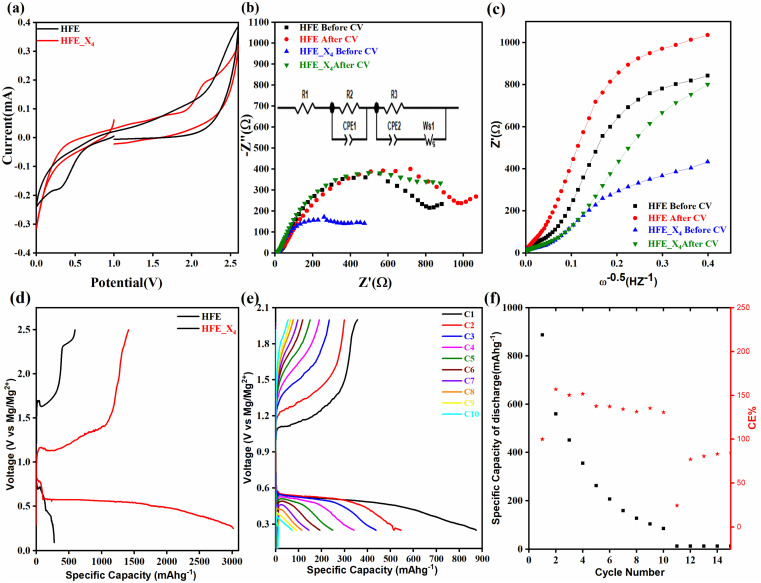
(a) CV curves at a scan rate of 8 mV s^−1^; (b) Nyquist plot and its equivalent circuit (inset); (c) linear fitting of Warburg impedance before and after cycling; (d) galvanostatic discharge–charge curves of HFE and HFE_X_4_; (e) galvanostatic discharge–charge curves of activated HFE_X_4_; (f) discharge capacity and coulombic efficiency *vs.* cycle number for activated HFE_X_4_.

**Fig. 6:**
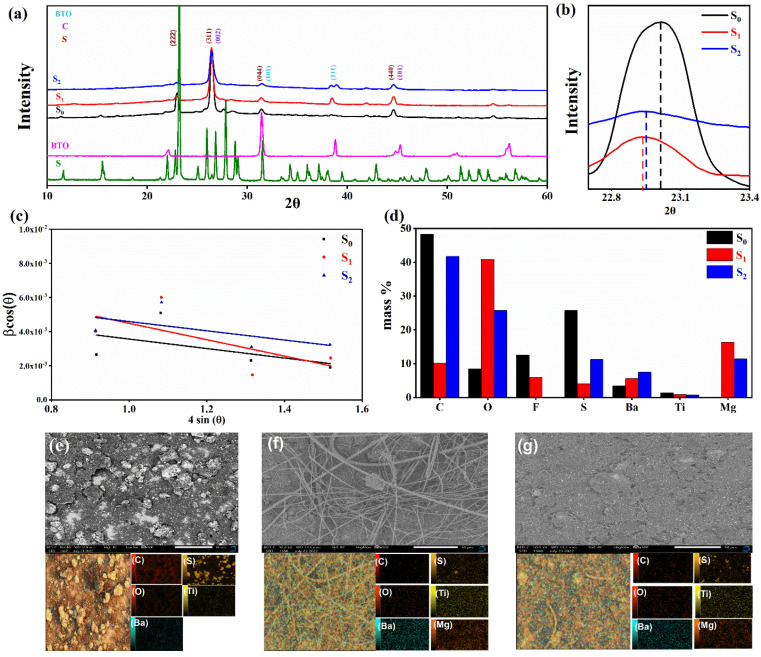
(a) The XRD pattern of the pristine sample cathode, after discharge and recharge; (b) zoom in 2*θ* = 22.5–23.5°; (c) the W–H analysis (d) EDS (elemental ratios); SEM micrographs and element mapping analysis spectra of (e) pristine cathode, (f) after Mg^2+^ insertion (g) after Mg^2+^ extraction.

Fig. 2c shows a Cole–Cole plot of the real dielectric constant *ε*′ *versus* imaginary dielectric loss *ε*′′ of HFE__*x*_EC electrolytes at 303 K, and each plot appears as a semicircle and intersects the *ε*′-axis. Relaxation time *τ* was calculated using the relation *τω*_max_ = 1, where *ω*_max_ is the angular frequency of the maximum *ε*′′. Fig. 2d shows that the relaxation time decreases exponentially with the temperature increase for all concentrations of EC due to the drop in the viscosity. Fig. 2e and f shows the combination of potentiostatic polarization and Cole–Cole plot of symmetrical Mg/HFE/Mg and Mg/HFE_X_4_/Mg cells. The values of the Mg^2+^ transference number *t*_Mg^2+^_ was calculated using Bruce–Vincent–Evans equation: 
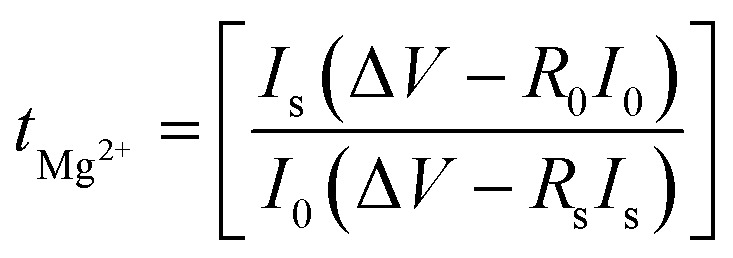
, where *I*_s_ and *I*_0_ are steady and initial state current, respectively, and Δ*V* is the applied voltage (0.01 V). The Mg-ion transference *t*_Mg^2+^_ values were recorded to be ∼0.77 and 0.866 for HFE, HFE_X_4_ electrolytes, respectively. The existence of the slope in the polarization curves is based on the reduction of the electrolyte with the high charge density electrode (Mg metal). Future work is in progress to protect the surface of the Mg anode from direct contact with the electrolyte solution or use functional additives to inhibit this reduction. The oxidative stability (−1–4.5 V (*vs.* Mg/Mg^2+^)) of the HFE and HFE_X_4_ electrolytes were measured by linear sweep voltammetry (LSV) in stainless-steel/HFE/Mg and stainless-steel/HFE_X_4_/Mg cells, Fig. 2g. A small oxidation peak at 3 V emerged in the plasticized electrolyte while the main oxidation potential onset of the plasticized electrolyte shifts to higher values >4 V compared with the pristine HFE, which suggests the role of EC in perturbing the bonding scheme of the pristine matrix. The evolution of the Mg electrolyte/electrode interface is systematically examined at rest using EIS. Fig. 3a and b show the Nyquist plots of Mg/HFE/Mg and Mg/HFE_X_4_/Mg cells with different rest time at open circuit voltage. The diameter of the semicircle decreases with an increase in the rest time, then decreases with a further increasing the rest time, and eventually stays at a stable value implying a stable interface between the electrolyte and the Mg-metal electrode. Fig. 3c shows Nyquist plots of symmetric Mg/HFE/Mg and Mg/HFE_X_4_/Mg cells before and after cycling. The plots were fitted using ZView software with the equivalent circuit shown as inset Fig. 3 and the obtained impedance parameters are listed in Table 1. EIS spectrum before/after cycling revealed a semicircle with an interface resistance (*R*_SEI_) of 34/78 Ω and a charge transfer resistance (*R*_ct_) of 10 089/8949 Ω in the pristine state. However, the cell with modified electrolyte (HFE_X_4_) possessed clear semicircles before discharge and open semicircle after discharge and significantly lower resistances before discharge and higher resistance after discharge compared with the cell with the pristine electrolyte which confirms that the modified electrolyte decomposed to a resistive SEI.^20,21^Fig. 3d shows the plating/stripping voltage profiles of symmetrical Mg/Mg cells using the HFE and HFE_X_4_ at 0.2 mA cm^−2^. Mg/HFE/Mg shows a stable voltage profile with low overpotential compared with symmetric Mg/HFE_X_4_/Mg cells, which show higher overpotential overgrowing with cycling. The results confirm that the EC reacts with the Mg anode during cycling, producing resistive byproducts that might have negatively affected the cycle life.^22^Fig. 4a–d shows SEM image and EDS results for bare Mg and Mg discs collected from disassembled Mg/HFE/Mg and Mg/HFE_X_4_/Mg symmetric cells after cycling. The surface of pristine Mg shows flat morphology, while that of Mg_HFE displays low-density stick morphology on the front-based Mg and the residual Si from the fiberglass separator. The surface of Mg_HFE_X_4_ shows high-density thick stick morphology compared with that of Mg/HFE. EDS analysis of bare Mg, Mg_HFE, and Mg/HFE_X_4_, as shown in Fig. 4c, shows that the spectral line intensity of Mg decreases from high in the bare Mg to 54.7 wt% in the Mg_HFE and further reduces to 24.87 wt% in the Mg/HFE_X_4_. The results confirm that the nature of the anode/electrolyte interface differs in the presence of EC, which reacts with Mg anode during cycling producing a passive byproduct as mentioned before. Fig. 4d shows the XRD diffraction pattern of bare Mg and Mg discs collected from disassembled Mg/HFE/Mg and Mg/HFE_X_4_/Mg symmetric cells after cycling. The reflection patterns in the three samples are the same and are centered on 2*θ* = 34.3°, 32.2°, 36.6°, 47.8°, and 63° assigned to the crystalline planes of (002), (100), (101), (102) and (103), respectively, [9, 10] of the hexagonal Mg structure (JCPDS No. 901-3058). To assess the electrochemical performance of the optimized electrolyte with the designed sulfur cathode, CV tests of Mg/HFE/S and Mg/HFE_HFE_X_4_/S cells were conducted in the potential range of 0–2.6 V at a scan rate of 8 mV s^−1^, Fig. 5a. The CV response shows that the cell with HFE_X_4_ has an observable anodic peak at ∼2 V and a higher cathodic/anodic current compared with the cell with HFE. However, this behavior confirms that electrolytes decomposed to an unstable SEI. EIS is regarded as one of the most powerful tools to analyze the cathode kinetic performance. The Nyquist plots of Mg/HFE/S and Mg/HFE_X_4_/S cells before and after short cycling in the frequency range between 1 Hz and 1 MHz are shown in Fig. 5b. The impedance spectra display intercept of *Z*′ at high frequency, semicircle at high-range frequency, semicircle at middle-range frequency, and a sloping line at low frequencies assigned to the bulk resistance of the electrolyte, diffusion, and migration of Mg^2+^ through the solid electrolyte interphase on the surface of the electrode (*R*_SEI_), charge transfer resistance (*R*_ct_) and to the Warburg impedance (represents magnesium-ion diffusion through the sulfur electrode), respectively. EIS parameters were calculated *via* fitting the equivalent circuit (Randell circuit model) using ZView software and the obtained parameters are displayed in Table 2. The diffusion coefficient of Mg^2+^ (DMg_^2+^_) was calculated by eqn:^23^
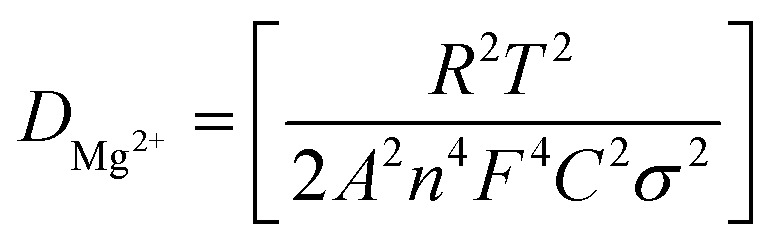
, from linear fitting the of the relation *Z*′ = *R*_s_ + *R*_ct_ + *σω*^−0.5^ (*ω*^−0.5^*vs. Z*′), the Warburg factor *σ* was calculated from the slope at low frequencies, Fig. 5c. The estimated values of *D*_Mg^2+^_ for all the cells are around 6 × 10^−15^ cm^2^ s^−1^. Fig. 5d shows the galvanostatic curves with a current density of 0.02 mA cm^−2^ for Mg/HFE/S and Mg/HFE_X_4_/S cells. The EC-containing electrolyte delivered a high discharge/charge capacity of ∼3000/1450 mA h g^−1^ over the theoretical capacity of the S electrode (∼1673 mA h g^−1^), while the pristine electrolyte (HFE) delivered reasonable discharge/charge below the theoretical capacity of sulfur with short cycle life. Thus, we can conclude that the two cells' major discharge/charge capacity value is due to the electrolyte decomposition. Aiming to improve the cycle life of the developed electrolyte in the Mg–S cells, galvanostatic cycling was considered as a conditioning process to scavenge the active contaminants and partially remove the passivation from the surface of the anode. Fig. 5e shows the voltage *vs.* specific capacity with a current density of 0.02 mA cm^−2^ of Mg/HFE_X_4_/S cell after pre-activating using the electrochemical conditioning processes. The activated cell delivered an initial discharge/charge capacity of ∼875/355 mA h g^−1^, and the cycle life extended to 10 cycles with coulombic efficiency >100%, suggesting that the electrolyte decomposition is still the major factor that controls the cycle life even with the electrochemical conditioning processes. The XRD was conducted to probe the crystal structure changes of the sulfur cathode from pristine → discharge → recharge states, Fig. 6a shows the XRD pattern of the sulfur, BTO powder, and sulfur cathode at different electrochemical states. XRD pattern of S_0_ (pristine cathode) shows the main peak locations at 23°, 25.74°, 28.58°, which matches the 222, 026, and 313 reflection signals of S_8_ with orthorhombic *S* lattice (DB Card No.: 9011362, Fddd: 2), confirming that sulfur is the main constituent of the cathode.^24^ After discharge, all peaks showed intense broadening indicating the formation of an amorphous electrolyte/cathode interface and the reaction of sulfur atoms with Mg^2+^. Fig. 6b shows the zoomed-in 2*θ* = 23° in the pristine state. The peak shifted to a low angle after discharge while it showed further reduction and a relatively a small shift to the original position after recharge indicates a partially reversible conversion reaction. The Williamson–Hall (W–H) model was applied to estimate the uniform stress deformation and the S cathode's average crystallite size (*D*) at different electrochemical states. The formula for W–H model is expressed as: 
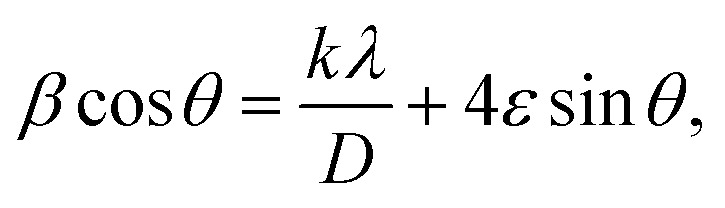
 where *β* is the full width at half maximum (FWHM), *θ* is the Bragg's angle, *k* is the shape factor (0.94), and *λ* is the X-ray wavelength. Fig. 6c reveals Williamson–Hall diagram: *β* cos(*θ*) *versus* 4 sin(*θ*) of S_0_, S_1,_ and S_2_, the slope of the line and the *y*-intercept represent the strain and crystallite size, respectively.^25^ The lattice strain values and crystallite size are listed in Table 3. The microstrain/crystallite size increases/decreases in the discharge state while returns to the original values upon after recharge state, which confirms that S cathode is under high volumetric expansion/contraction during the insertion/extraction of Mg^2+^ within the sulfur skeleton. SEM images and EDS results of the S_0_, S_1_, and S_2_ composite are shown in Fig. 6d–g. The pristine S_0_ electrode shows an inhomogeneous distribution of sulfur deposits on the GNPs network surface. After magnesiation (S_1_), it can be noticed that the Mg^2+^ ions interact with S atoms and show stick sediment distribution. The stick sediments disappeared in de-magnesiation state (S_2_), and the surface showed smooth morphology. Fig. 6d and insets of Fig. 6e–g show EDS and elemental mapping results for S_0_, S_1_, and S_2_, respectively. By following the evolution of Mg^2+^ ratio that increased from zero in the pristine state to 16.32 wt% upon discharge and decreased to 11.48 wt% upon recharge state, the results support the partially reversible conversion reaction of the S_8_ to polysulfide molecules and *vice versa*. Furthermore, the evolution of the sulfur ratio from 18 wt% in S_2_ after discharging to 6 wt% upon subsequent charge process confirms the shuttling of polysulfide, which is presumably responsible for the capacity fading.

**Table 1:** EIS parameters of symmetric Mg/Mg cells with HFE and HFE_X_4_ electrolyte before cycling and after 70 cycles

EIS parameters	*R*_s_ (Ω)	*R*_SEI_ (Ω)	*R*_ct_ (Ω)
HFE before cycling	24.56	43	10 089
HFE after cycling	33.58	78.6	8949
HFE_X_4_ before cycling	21.72	56	6960
HFE_X_4_ after cycling	41.21	112.81	13 400

**Table 2:** Electrochemical impedance parameters of electrodes

EIS parameters	*R*_s_ (Ω)	*R*_SEI_ (Ω)	*R*_ct_ (Ω)	CPE (*F* × 10^−4^)	Aw (Ω)	*D*_Mg^2+^_ [cm^2^ s^−1^]
0%	14.93	39.93	866.3	0.99743	648.48	6.18 × 10^−15^
20%	13.95	34.93	533.4	0.30603	652.3	6.1 × 10^−15^

**Table 3:** Crystallite size and strain values for the cathode at different electrochemical states

Element	Pristine	Mg^2+^ insertion	Mg^2+^ extraction
Strain (*ε*) ×10^−3^	2.78	4.8	2.7
Crystallite size D (nm)	22.84	15.61	19.87

## Conclusion

In this work, we investigated the efficiency and electrochemical compatibility of EC additive to halogen-free electrolyte based Mg(NO_3_)_2_ in acetonitrile (ACN) and tetraethylene glycol dimethyl ether (G4). The FT-IR spectra confirm that EC additive changed the Mg^2+^ solvation structure *via* supporting weak solvent–cation interaction, promoting the rapid desolvation of Mg^2+^ ions. Furthermore, the addition of EC enhanced the ionic conductivity and Mg^2+^ ion transference number of the electrolyte, which can be understood from the role of EC in disassociating the magnesium salt and forming SEI. However, the introduction of EC results in the formation of a resistive SEI, causing high Mg stripping/plating overpotential. The Mg–S full cell using modified HFE (HFE_EC) delivers an initial specific capacity of 900 mA g^−1^ with a cycle life of up to 10 cycles in case of activating the cell with electrochemical conditioning. Much work must be dedicated to optimizing the EC-based halogen-free electrolytes to realize practical Mg–S batteries.

## Conflicts of interest

There are no conflicts to declare.

## Supplementary Material
